# FGF21 promotes thermogenic gene expression as an autocrine factor in adipocytes

**DOI:** 10.1016/j.celrep.2021.109331

**Published:** 2021-06-29

**Authors:** Mohammad Abu-Odeh, Yuan Zhang, Shannon M. Reilly, Nima Ebadat, Omer Keinan, Joseph M. Valentine, Maziar Hafezi-Bakhtiari, Hadeel Ashayer, Lana Mamoun, Xin Zhou, Jin Zhang, Ruth T. Yu, Yang Dai, Christopher Liddle, Michael Downes, Ronald M. Evans, Steven A. Kliewer, David J. Mangelsdorf, Alan R. Saltiel

**Affiliations:** 1 Department of Medicine, University of California, San Diego, San Diego, CA 92093, USA; 2 Department of Pharmacology, UT Southwestern Medical Center, Dallas, TX 75390, USA; 3 Department of Pharmacology, University of California, San Diego, San Diego, CA 92093, USA; 4 Moores Cancer Center at UC San Diego Health, La Jolla, CA 92037, USA; 5 Department of Bioengineering, University of California San Diego, San Diego, CA 92093; 6 Department of Chemistry and Biochemistry, University of California San Diego, San Diego, CA 92093, USA; 7 Gene Expression Laboratory, Salk Institute for Biological Studies, La Jolla, CA 92037, USA; 8 Storr Liver Centre, Westmead Institute for Medical Research and Sydney Medical School, University of Sydney, Westmead, NSW, Australia; 9 Department of Molecular Biology, UT Southwestern Medical Center, Dallas, TX 75390, USA; 10 Howard Hughes Medical Institute; 11 Lead contact

## Abstract

The contribution of adipose-derived FGF21 to energy homeostasis is unclear. Here we show that browning of inguinal white adipose tissue (iWAT) by β-adrenergic agonists requires autocrine FGF21 signaling. Adipose-specific deletion of the FGF21 co-receptor β-Klotho renders mice unresponsive to β-adrenergic stimulation. In contrast, mice with liver-specific ablation of FGF21, which eliminates circulating FGF21, remain sensitive to β-adrenergic browning of iWAT. Concordantly, transgenic overexpression of FGF21 in adipocytes promotes browning in a β-Klotho-dependent manner without increasing circulating FGF21. Mechanistically, we show that β-adrenergic stimulation of thermogenic gene expression requires FGF21 in adipocytes to promote phosphorylation of phospholipase C-γ and mobilization of intracellular calcium. Moreover, we find that the β-adrenergic-dependent increase in circulating FGF21 occurs through an indirect mechanism in which fatty acids released by adipocyte lipolysis subsequently activate hepatic PPARα to increase FGF21 expression. These studies identify FGF21 as a cell-autonomous autocrine regulator of adipose tissue function.

## In brief

Abu-Odeh et al. demonstrate that autocrine action of FGF21 is a required second signal promoting thermogenic gene expression in catecholamine-stimulated adipocytes. Hepatic FGF21 secretions, secondary to catecholamine-stimulated adipocyte lipolysis, are dispensable for adipose tissue browning. These studies identify FGF21 as a cell-autonomous autocrine regulator of adipose tissue function.

## Graphical Abstract

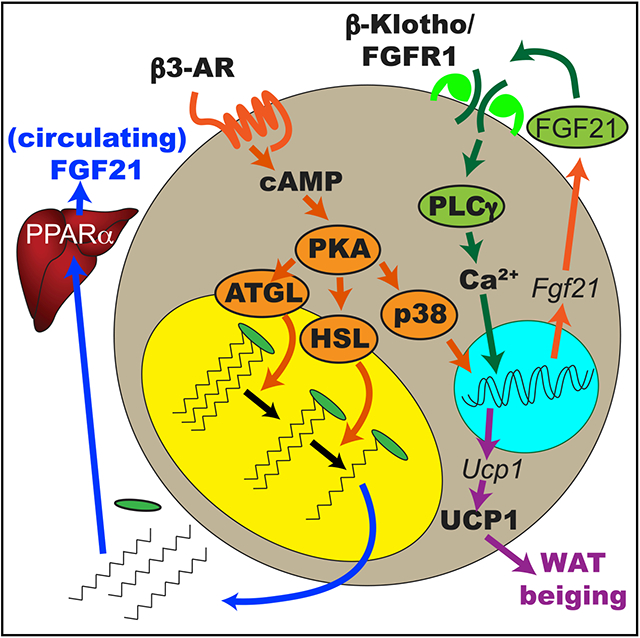

## INTRODUCTION

FGF21 is a member of the fibroblast growth factor (FGF) superfamily of growth factors (Beenken and Mohammadi, 2009). Like FGF19 and FGF23, FGF21 is an atypical member of this superfamily that lacks a heparin-binding domain and is released into the blood-stream to act as a hormone. FGF21 is synthesized by several organs, where it is induced by various states of stress, including cold and fasting (BonDurant and Potthoff, 2018; Fisher and Maratos-Flier, 2016; Kharitonenkov and DiMarchi, 2017; Kliewer and Mangelsdorf, 2019). FGF21 exerts its effects by binding to a heteromeric receptor comprised of FGF receptor 1c (FGFR1c) and the obligate co-receptor β-Klotho (BonDurant and Potthoff, 2018; Fisher and Maratos-Flier, 2016; Kharitonenkov and DiMarchi, 2017; Kliewer and Mangelsdorf, 2019). Among its downstream actions, FGF21 regulates energy metabolism. Exogenous administration of FGF21 to obese mice induces weight loss, corrects hyperlipidemia, reduces inflammation, lowers blood sugar and insulin resistance, and improves fatty liver.

FGF21 is induced by cold exposure in white and brown adipose tissue (WAT and BAT, respectively) and liver and contributes to maintaining body temperature (Ameka et al., 2019; Fisher et al., 2012; Hondaresetal., 2011). An important component of increased thermogenesis involves “browning” or “beiging,” in which brown-like, uncoupling protein 1 (UCP1)-expressing adipocytes emerge in inguinal WAT (iWAT) depots (Cohen and Spiegelman, 2015). FGF21 induces efficient browning of iWAT (Fisher et al., 2012). Mice lacking FGF21 are impaired in their ability to adapt to chronic cold exposure and have an accompanying reduction in browning of WAT. FGF21 increases browning of WAT through a two-pronged mechanism. First, it acts centrally to activate the sympathetic nervous system (Ameka et al., 2019; Douris et al., 2015; Owen et al., 2014). Second, it acts directly on white adipocytes to induce thermogenic gene expression (Fisher et al., 2012).

The mechanism by which FGF21 promotes WAT browning has been the subject of much debate. Although FGF21 levels are increased in response to cold exposure, there has been much uncertainty about the tissues that secrete FGF21, the signaling events involved in control of expression and secretion of the hormone, its site of action, and the important signaling pathways used by the hormone to elicit its beneficial effects. Prior studies have suggested autocrine actions of FGF21 in adipocytes (Fisher et al., 2012; Justesen et al., 2020). However, the physiological relevance of autocrine FGF21 activity in adipose tissue remains uncertain, and the mechanism by which the hormone elicits its effects is unknown.

Here we explore the role of FGF21 in browning of iWAT using the synthetic β−3 adrenergic agonist CL-316,243. We also examine the tissue sources of FGF21 and the signaling pathway it activates in white adipocytes to promote iWAT browning. These studies reveal that many of the *in vivo* beneficial effects of FGF21 arise from its actions as an autocrine/paracrine factor in fat that delivers a necessary second signal for full expression of the thermogenic program.

## RESULTS

### FGF21 is produced in response to β−3 adrenergic activation in multiple tissues

**Figure 1.**
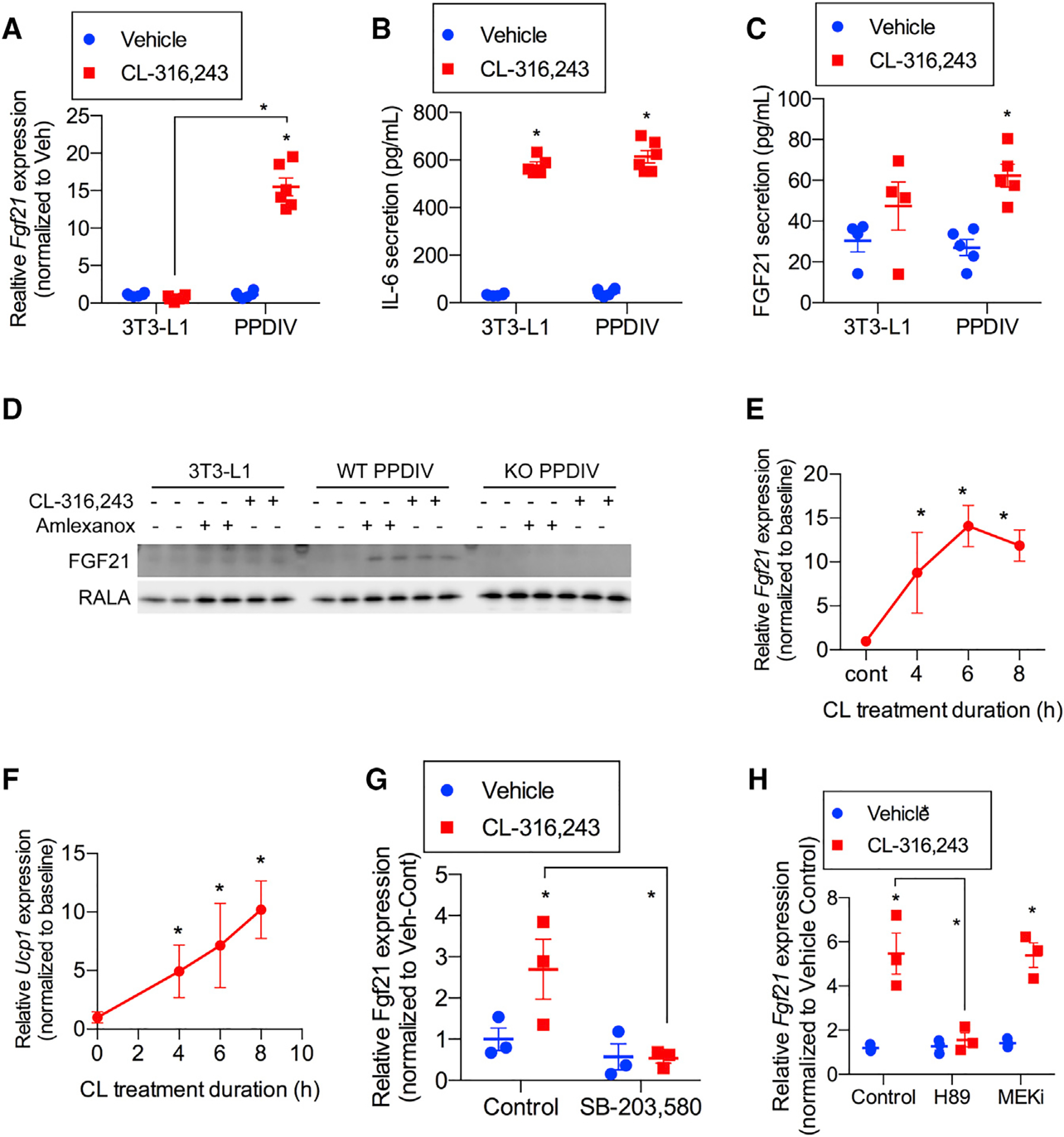
Fgf21 mRNA expression and secretion are induced in PPDIVs following CL-316,243 (A) *Fgf21* mRNA expression in 3T3-L1 adipocyte and PPDIVS following 10 μM CL-316,243 treatment for 6 h. n = 4–6 wells per cell type per treatment. (B and C) IL-6 (B) and FGF21 (C) secretion following treatment with CL-316,243 as in (A). n = 4–6 wells per cell type per treatment. (D) Western blot analysis of FGF21 protein levels in 3T3-L1, PPDIVs isolated from WT mice, and PPDIVs isolated from whole-body FGF21-KO. n = 2 wells per genotype/cell type per treatment. (E and F) *Fgf21* (E) and *Ucp1* (F) mRNA time course expression in WT PPDIVs following 10 μM CL-316,243 treatment. n = 3 wells per time point. (G and H) *Fgf21* mRNA expression in cells pretreated with 10 μM SB-303,580 (p38 inhibitor) (n = 3 wells per treatment) (G) and 50 μM H89 (PKA inhibitor) or 10 μM MEK inhibitor (PD0325901) (H) for 30 min before 10 μM CL-316,243. n = 3 wells per genotype per treatment. Data presented as mean ± SEM. (A–C, G, and H) *p < 0.05 from Holm-Sidak post hoc analysis after significant two-way ANOVA for vehicle versus CL-316,243 unless otherwise indicated with a line. (E and F) *p < 0.01 from t test versus baseline.

Although several studies suggest that circulating FGF21 is largely produced by the liver (Ameka et al., 2019; Liang et al., 2014; Markan et al., 2014), its mRNA is also expressed in other tissues, where it might act as an autocrine or paracrine factor. One such tissue is adipose tissue; FGF21 expression is induced in response to β-adrenergic agonists in BAT and WAT in mice (Chartoumpekis et al., 2011; Peng et al., 2015). We sought to understand the role of FGF21 in adipocytes. Because fasting increases sympathetic outflow to iWAT (Brito et al., 2008), we treated 3T3-L1 adipocytes and primary preadipocytes differentiated *in vitro* (PPDIVs) from subcutaneous WAT with the β−3 adrenergic agonist CL-316,243 and assessed *Fgf21* mRNA and protein secretion into the medium (Figure 1). *Fgf21* mRNA was not increased in 3T3-L1 adipocytes in response to CL-316,243 (Figure 1A), although the agonist dramatically increased interleukin-6 (IL-6) production in these cells (Figure 1B). In contrast, CL-316,243 produced a marked increase in *Fgf21* mRNA in PPDIVs (Figure 1A). FGF21 protein was also secreted into the medium in these experiments (Figure 1C), and protein was detected in lysates from PPDIVs but not 3T3L1 adipocytes (Figure 1D). *Fgf21* mRNA was induced in a time-dependent manner following treatment of PPDIVs with CL-316,243, for 4–8 h (Figure 1E). Moreover, uncoupling protein (*Ucp1*) mRNA was also induced by CL 316,243 (Figure 1F).

We assessed the pathways responsible for increasing *Fgf21* mRNA expression in adipocytes. Binding of CL-316,243 to β−3 adrenergic receptors is known to activate adenylyl cyclase and increase levels of cyclic AMP (cAMP) (Collins, 2012; Mirbolooki et al., 2014). When generated, cAMP can activate cAMP-dependent protein kinase A, which, in turn, leads to activation of the mitogen-activated protein kinase (MAPK) p38 and phosphorylation of transcription factors such as activating transcription factor 4 and cAMP response element-binding protein, controlling gene expression (Tchivileva et al., 2009). Stimulation of *Fgf21* mRNA production by CL-316,243 was prevented by preincubating PPDIVs with inhibitors of p38 (SB-203,580) (Zhen et al., 2001) or protein kinase A (PKA) (H89) (Limbutara et al., 2019; Figures 1G and 1H) but not with the MAPK/ERK kinase (MEK) inhibitor PD98059 (Dudley et al., 1995; Figure 1H), demonstrating that regulation of FGF21 production is downstream of the PKA/p38 axis, consistent with previous findings in brown fat (Hondares et al., 2011).

### Circulating FGF21 is derived exclusively from the liver in mice treated with adrenergic agonists because of stimulation of adipocyte lipolysis

**Figure 2.**
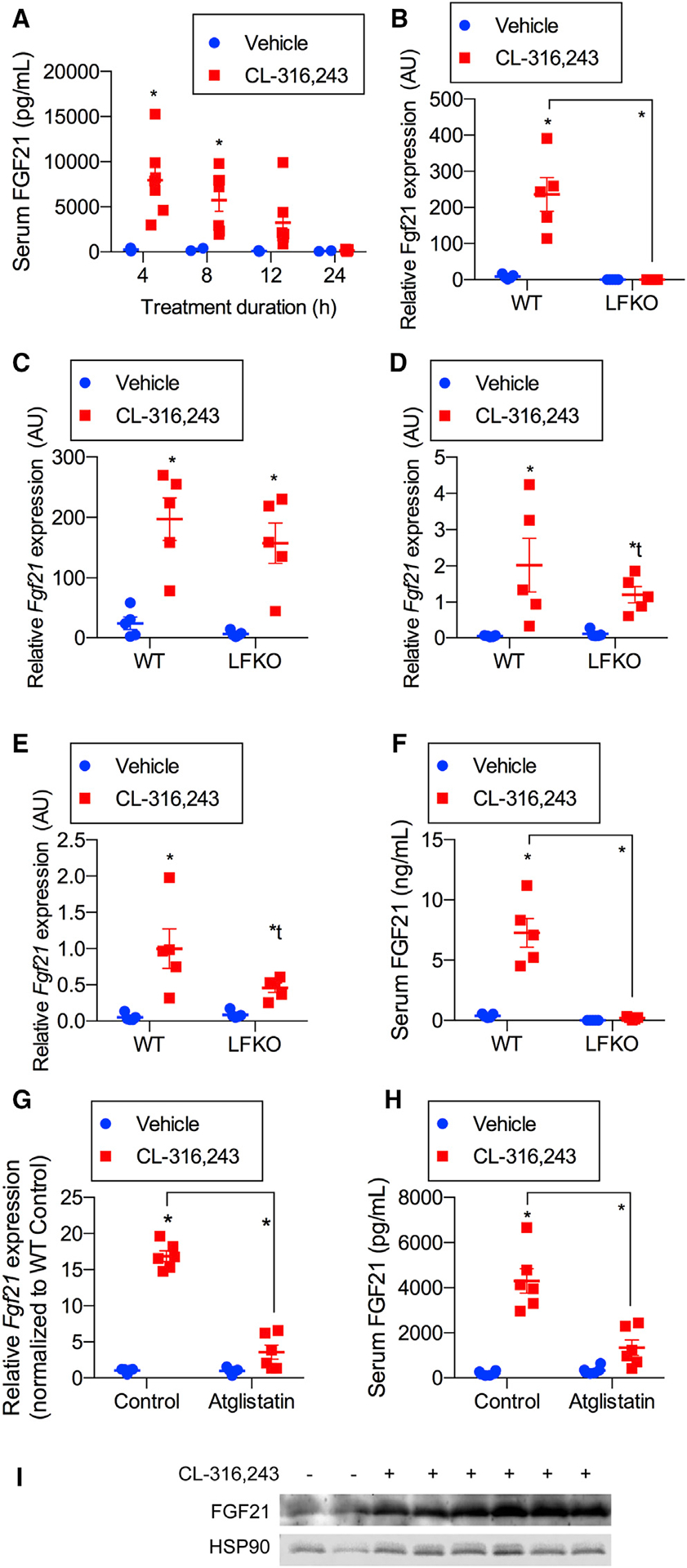
Circulating FGF21 is derived exclusively from the liver in mice treated with adrenergic agonists because of stimulation of adipocyte lipolysis (A) Serum FGF21 time course following intraperitoneal injection of 1 mg/kg CL-316,243 or vehicle (0, 4 h, 8 h, 12 h, and 24 h). n = 6 CL-316,243 (CL) and 3 vehicle-treated animals per time point. (B–E) WT-mice and LFKO (liver-specific *Fgf21* KO) mice treated with 1 mg/kg CL or vehicle treatment. n = 5 mice per genotype per treatment. *Fgf21* mRNA expression after 4-h treatment in (B) the liver, (C) brown adipose tissue (BAT),(D) inguinal WAT (iWAT), and (E) epididymal WAT (eWAT). (F) Serum FGF21 4 h after treatment. (G and H) Liver *Fgf21* mRNA expression (G) and serum FGF21 (H) from ageand weight-matched mice pretreated with 50 μM atglistatin for 30 min, followed by 1 mg/kg CL treatment for 4 h. n = 6 animals per treatment group. (I) Western blot analysis of FGF21 protein from protein lysates isolated from iWAT after treating the mice as in (A) for 4 h. n = 2 vehicle-treated and 6 CL-treated mice. Data presented as mean ± SEM. *p < 0.05 from Holm-Sidak post hoc analysis after significant two-way ANOVA for vehicle versus treated group unless otherwise indicated with a line.

Although previous studies showed that most of the circulating FGF21 elevated during fasting is derived from the liver (BonDurant and Potthoff, 2018; Nishimura et al., 2000), the source of the hormone produced during sympathetic activation remains uncertain. Some studies have implicated adipocytes in FGF21 secretion after cold exposure (Hondares et al., 2011), whereas others have demonstrated that hepatic FGF21 secretion determines serum levels (Ameka et al., 2019). Thus, we injected mice with CL-316,243 or vehicle and analyzed FGF21 expression for 4–24 h (Figure 2A). Interestingly, FGF21 concentrations were maximal in the blood after 4 h and declined thereafter. This time course was consistent with immediate increases in *Fgf21* mRNA in the liver (Figure 2B) and all adipose depots (Figures 2C–2E). To determine the source of FGF21 in the circulation, we crossed floxed-*Fgf21* with albumin-Cre mice to generate hepatocyte-specific knockout (liver-specific FGF21 knockout [LFKO]) of the hormone (Lan et al., 2017; Markan et al., 2014). Wild-type (WT) and conditional KO mice were then treated with CL-316,243, and serum levels of FGF21 were assayed 4 h after the injections (Figure 2F). The β−3-adrenergic agonist produced a significant increase in circulating levels of the hormone in WT mice, but this response was ablated in the LFKO mice despite dramatic increases in mRNA levels in all adipose depots (Figures 2C–2E). These data confirm that the vast majority of circulating FGF21 induced by *in vivo* β−3-adrenergic agonist treatment is derived from hepatocytes, consistent with what has been observed upon cold exposure (Ameka et al., 2019).

Because the β−3-adrenergic receptor is expressed primarily in adipocytes and to a lesser extent in the bladder, it is unlikely that CL-316,243 directly increased expression of *Fgf21* mRNA in the liver. We thus wanted to find out whether this increase might be indirect because of activation of lipolysis in adipocytes. This is consistent with the observation that *Fgf21* is a target gene of the nuclear fatty acid receptor PPARa (Badman et al., 2007; Inagaki et al., 2007, 2008). To address this possibility, we treated mice with the lipolysis inhibitor atglistatin (Mayer et al., 2013) prior to injection with CL-316,243 (Figure 2G; Figure S1A). Atglistatin pretreatment dramatically reduced stimulation of lipolysis by CL-316,243 injection (Figure S1B) and also prevented induction of hepatic *Fgf21* mRNA (Figure 2G) and circulating FGF21 (Figure 2H) with no effect on *Fgf21* gene expression in WAT (Figure S1C). We also demonstrated that direct treatment of primary hepatocytes with the β-adrenergic agonist isoproterenol or CL-316,243 (Figure S1D) did not increase FGF21 secretion despite a dramatic increase in cAMP levels seen with glucagon in these cells (Figure S1E). These data indicate that *Fgf21* gene expression in the liver is independent of direct β-adrenergic signaling and dependent on activation of PPARα via increased fatty acids derived from adipocytes.

Although the source of circulating FGF21 produced in response to adrenergic activation can be ascribed to the liver, we sought to determine whether the hormone is synthesized at appreciable levels in adipose tissues *in vivo*. Western blot analyses of adipose tissue depots 4 h after CL-316,243 injection revealed that FGF21 protein is readily detected in adipose tissues (Figure 2I). The observation that the protein is produced in adipocytes *in vitro* and *in vivo* but does not appear to escape into the circulation suggests that FGF21 may play an autocrine or paracrine role in adipose tissues.

### The metabolic benefit of FGF21 is due to its action as an autocrine factor in iWAT

**Figure 3.**
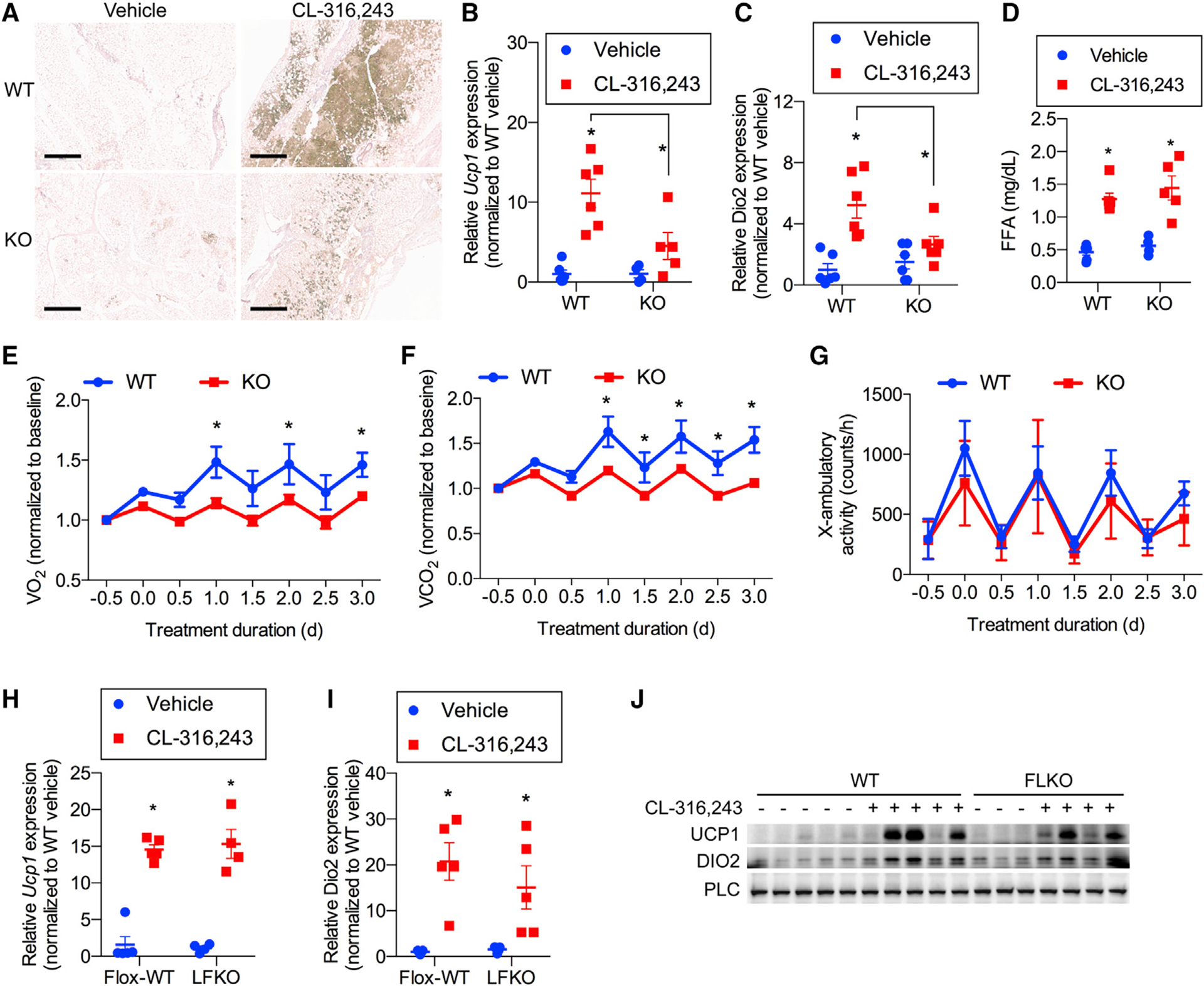
The metabolic benefit of FGF21 is due to its action as an autocrine factor in iWAT (A–C) WT and whole-body FGF21-KO mice treated with 1 mg/kg CL or vehicle daily for 7 days. n = 6 mice per genotype per treatment. (A) Representative immunohistochemistry (IHC) staining of UCP1 in iWAT. Scale bar, 0.5 mM. (B and C) *Ucp1* (B) and *Dio2* (C) mRNA expression in iWAT. (D) Serum free fatty acid (FFA) concentrations 20 min after treatment with 1 mg/kg CL or vehicle in WT and FGF21-KO mice. n = 6 mice per genotype per treatment. (E–G) Baseline normalized oxygen consumption (E), baseline normalized carbon dioxide production (F), and physical activity (G) as assayed by x axis beam breaks in WT and whole body FGF21-KO mice treated with 1 mg/kg CL daily. n = 6 WT and 7 KO mice. (H and I) *Ucp1* (H) and *Dio2* (I) mRNA expression of WT mice and LFKO mice treated with 1 mg/kg CL or vehicle daily for 7 days. n = 5 mice per genotype per treatment. (J) Western blot analysis of FGF21 protein in iWAT following 7-day treatment with 1 mg/kg CL or vehicle in WT and LFKO mice. n = 5 WT mice per treatment group and 3 vehicle-treated and 4 CL-treated FLKO mice. Data presented as mean ± SEM.*p < 0.05 from Holm-Sidak post hoc analysis after significant two-way ANOVA for vehicle versus CL unless otherwise indicated with a line.

Thermogenic beige adipocytes appear in white adipose depots after prolonged cold exposure or activation of β-adrenergic receptors via induction of the uncoupling protein UCP1 and other thermogenic genes (Cohen and Spiegelman, 2015). To test whether FGF21 is required for browning of iWAT in response to β-adrenergic receptor agonism, we treated whole-body FGF21 KO mice and their WT littermates with a 7-day regimen of CL-316,243 by daily injection (Figure 3). CL-316,243 stimulated browning of iWAT in WT mice, as evidenced by immunohisto-chemical staining for UCP1 (Figure 3A) and induction of *Ucp1* and *Dio2* mRNA (Figures 3B and 3C). No effect was observed in BAT (Figure S2). Interestingly, all effects of β-adrenergic stimulation on browning of iWAT were reduced in FGF21-KO mice (Figures 3A–3C). This result is consistent with prior observations that FGF21-KO mice display defects in cold-induced WAT browning (Fisher et al., 2012). Importantly, this was not due to generalized catecholamine resistance because the lipolytic response to CL-316,243 treatment was equal in both genotypes (Figure 3D). To determine whether the observed defect in WAT beiging had a significant effect on whole-body energy expenditure, we performed a metabolic cage study of WT and FGF21-KO mice treated with CL-316,243 (Figure 3E–G). CL-316,243 treatment significantly increased dark-cycle oxygen consumption and carbon dioxide production in WT but not FGF21-KO mice (Figures 3E and 3F), suggesting that the beiging defect in FGF21-KO mice translates to a defect in CL-316,243-stimulated energy expenditure in these mice. No difference in physical activity was observed between the two genotypes (Figure 3G). As observed previously (Badman et al., 2009), FGF21-KO mice tended to have a lower body weight than their WT littermate controls (Figure S3A). However, the 1-week treatment period with CL-316,243 was not sufficient to induce significant weight loss (Figure S3A). Both genotypes displayed the same degree of reduction in fasting blood glucose (Figure S3B), and no differences in serum insulin levels were observed (Figure S3C).

Because CL-316,243 increased FGF21 expression in multiple tissues, we wondered whether the browning of iWAT is due to local or circulating increases in FGF21. To this end, we injected flox-WT and LFKO mice with CL-316,243 daily for 1 week (Figures 3H–3J). LFKO mice have been shown previously to exhibit small defects in glucose handling and core temperature maintenance in response to acute cold exposure (Ameka et al., 2019; Markan et al., 2014). Interestingly, despite the lack of circulating FGF21 in LFKO mice (Figure 2C), increased *Ucp1* and *Dio2* expression and UCP1 protein levels in iWAT were identical in both genotypes (Figures 3H–3J). This result demonstrates that circulating, liver-derived FGF21 is dispensable for browning of iWAT, indicating that the hormone is likely to behave as an autocrine factor in adipose tissue.

**Figure 4.**
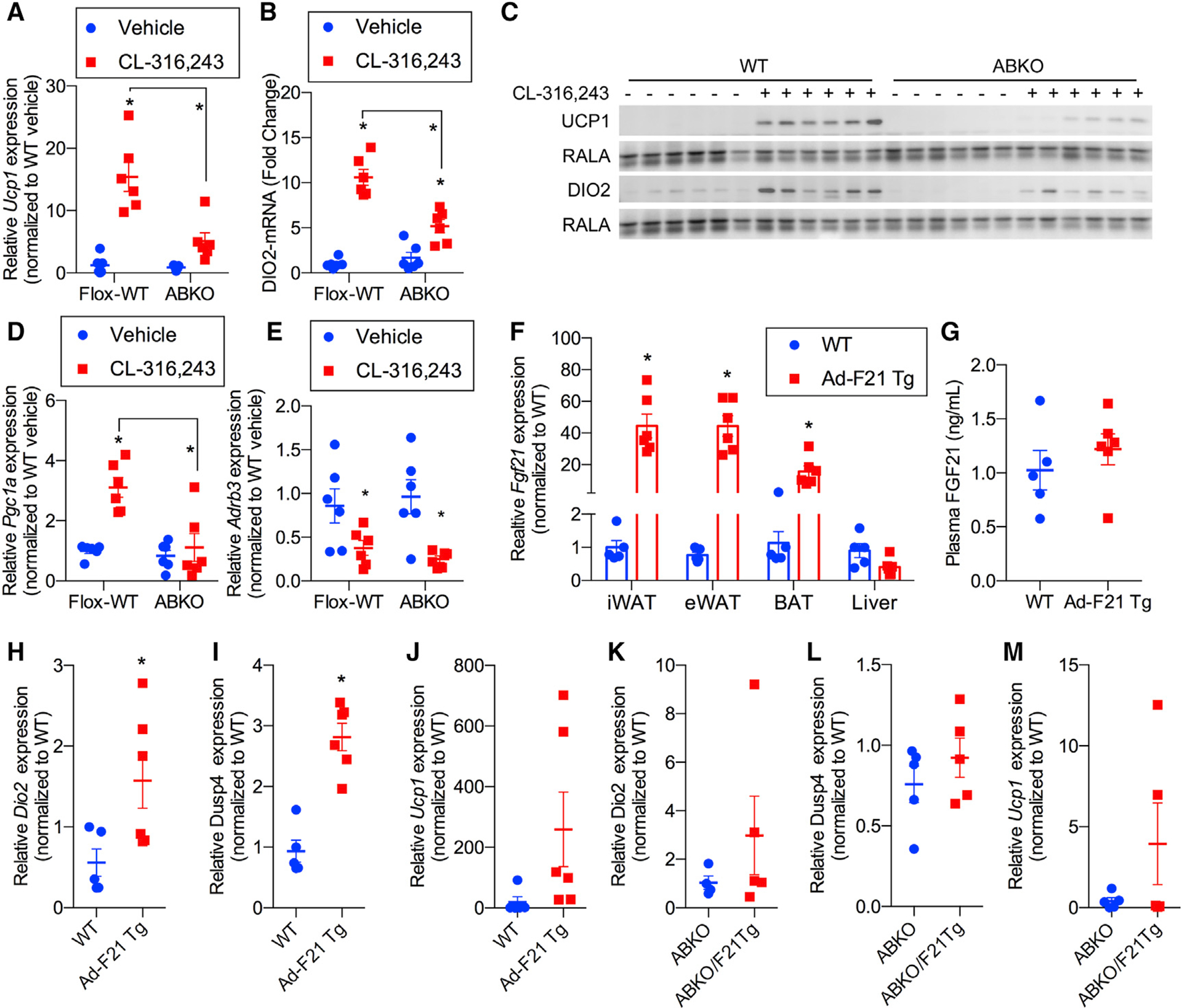
FGF21 operates in an autocrine fashion by binding to its receptor in adipocytes (A and B) *Ucp1* (A) and *Dio2* (B) mRNA expression in iWAT from WT (flox-WT) and ABKO mice treated with 1 mg/kg CL or vehicle daily for 7 days. n = 6 mice per genotype per treatment. (C) Western blot analysis of UCP1, anti DIO2, and RalA (as a loading control) protein levels in iWAT from mice in (A). n = 6 mice per genotype per treatment. (D and E) *Pgc1a* (D) and *Adrb3* (E) mRNA expression in iWAT isolated from WT and ABKO mice treated with 1 mg/kg CL or vehicle daily for 7 days. n = 6 mice per genotype per treatment. (F–J) Ad-F21 Tg and littermate WT controls. n = 5–6 mice per genotype. (F) *Fgf21* mRNA expression in adipose tissues and liver. (G) Plasma FGF21 protein levels measured by ELISA. (H–J) *Dio2* (H), *Dusp4* (I), and *Ucp1* (J) mRNA expression in iWAT. (K–M) *Dio2*, *Dusp4*, and *Ucp1* mRNA expression in iWAT isolated from ABKO and ABKO/F21 Tg mice. n = 5–6 mice per genotype. Data presented as mean ± SEM.*p < 0.05 from Holm-Sidak post hoc analysis after significant two-way ANOVA for vehicle versus CL unless otherwise indicated with a line.

To explore the direct action of FGF21 on adipocytes, we utilized mice selectively lacking the obligate FGF21 co-receptor β-Klotho in adipose tissue by crossing β-Klotho floxed with adiponectin-Cre mice (adipose-specific β-Klotho KO [ABKO]), which do not exhibit an overt metabolic phenotype at baseline (Lan et al., 2017). We treated floxed and ABKO mice with daily injections of CL-316,243 for 1 week (Figures 4A–4E). Similar to what we observed in the FGF21-KO animals, ABKO mice were defective in their ability to increase expression of the thermogenic genes *Ucp1* and *Dio2* in WAT in response to CL-316,243 treatment (Figures 4A and 4B). Corresponding UCP1 and DIO2 protein levels were induced significantly in flox-WT controls but not ABKO mice (Figure 4C). CL-316,243-induced expression of the browning-associated gene *Pgc1a* (Figure 4D) was also attenuated in ABKO mice. Importantly, no differences were observed between the genotypes regarding the effect of CL-316,243 on downregulation of the β−3-adrenergic receptor *Adrb3* (Figure 4E), as shown previously (Ferrand et al., 2006), suggesting that FGF21 action is required for only a subset of genes induced by β-adrenergic receptor activation.

To further explore the likelihood that FGF21 operates in an autocrine fashion in adipocytes, we utilized a mouse model with adipocyte-specific transgenic overexpression of FGF21 (Ad-F21 Tg) (Figure S4A). *Fgf21* mRNA levels were increased in all adipose depots, whereas liver *Fgf21* expression remained unchanged (Figure 4F). Consistent with the lack of effect of FGF21 on adipocyte lipolysis, we did not observe any changes in expression of genes encoding the key lipolytic enzymes *Atgl* and *Lipe* nor a change in serum lipid levels (Figures S4B–S4D). As expected, adipocyte-specific FGF21 expression did not have any effect on circulating FGF21 levels (Figure 4G). Nevertheless, adipocyte-expressed FGF21 was sufficient to upregulate expression of *Dio2* and *Dusp4*, both of which are associated with WAT browning (Figures 4H and 4I). There was a trend toward upregulation of Ucp1 (Figure 4J). Thus, it is not clear whether adipocyte FGF21 is sufficient to upregulate *Ucp1* expression. One possibility is that FGF21 is necessary but not sufficient for induction of UCP1 expression in white adipocytes, suggesting an obligate two-signal mechanism where β-adrenergic signaling through cAMP and FGF21 signaling is required for UCP1 upregulation in white adipocytes. Further supporting an autocrine function of adipocyte-secreted FGF21, the adipocyte-specific transgene did not increase expression of these genes in adipocyte-specific β-Klotho KO mice (Figures 4K–4M). These data indicate that FGF21 is produced in adipocytes in response to sympathetic activation and works locally to support expression of thermogenic genes.

### FGF21 provides a second signal that is required for browning of WAT

**Figure 5.**
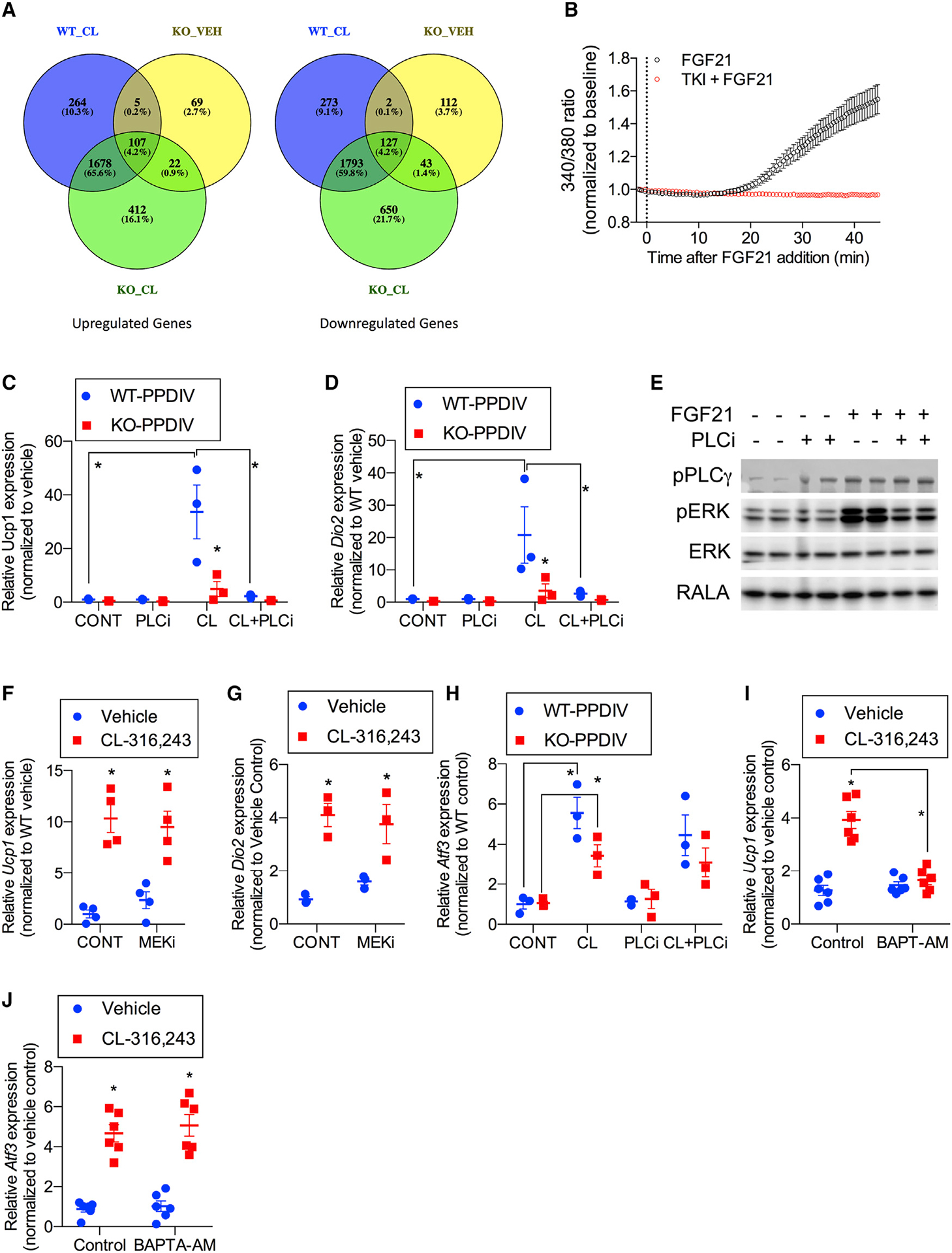
FGF21 provides a second signal that is required for browning of WAT Genes involved in calcium signaling are upregulated by CL in a FGF21-dependent manner. (A) Venn diagrams of differentially expressed genes from RNA-seq analysis of WT and FGF21-KO PPDIVs treated with vehicle or CL (Data S1). n = 3 wells per treatment per genotype. (B) Effect of FGF21 on calcium mobilization in the absence or presence of 200 nM tyrosine kinase inhibitor (PD173074). PPDIVs were serum starved for 15 h and loaded with fura-2 AM for 15 min in the absence (black trace, n = 63 cells) or presence (red trace, n = 23 cells) of PD173074 before stimulation with FGF21 (200 ng/mL). Curves are pooled from 4 and 2 experiments, respectively. Values are mean ± SEM. (C and D) *Ucp1* (C) and *Dio2* (D) mRNA expression in PPDIVs isolated from FGF21-WT mice or FGF21-KO mice; cells were pretreated with 1 μM of PLC inhibitor (U73122) for 30 min, followed by 10 μM CL treatment for 6 h. n = 3 wells per genotype per treatment. (E) Western blot analysis of PPDIVs pretreated with 1 μM PLC inhibitor (U73122) for 30 min, followed by 100 ng/mL recombinant FGF21 for 10 min. n = 2 wells per treatment. The membrane was blotted against antibodies as indicated. (F and G) *Ucp1* (F) and *Dio2* (G) expression in PPDIVs pretreated with 10 μM MEK inhibitorPD0325901 (MEKi) for 30 min, followed by 10 μM CL treatment for 6 h. n = 3–4 wells per cell type per treatment. (H) *Atf3* mRNA expression in PPDIVs isolated from FGF21-WT mice or FGF21-KO mice pretreated with 1 μM of PLC inhibitor (U73122) for 30 min, followed by 10 μM CL treatment for 6 h. (I and J) *Ucp1* (I) and *Atf3* (J) mRNA expression in PPDIVs isolated from WT mice. Following differentiation, the cells were pretreated with 10 μM BAPTA-AM for 30 min, followed by 10 μM CL treatment for 6 h. n = 6 wells per cell type per treatment. Data presented as mean ± SEM.*p < 0.05 from Holm-Sidak post hoc analysis after significant two-way ANOVA for vehicle versus CL unless otherwise indicated with a line.

To shed more light on the FGF21 signaling pathway responsible for inducing thermogenic gene expression in adipocytes, we performed RNA sequencing (RNA-seq) using PPDIVs isolated from WT and whole-body FGF21-KO mice treated with or without CL-316,243 after differentiation (Figure 5A; Data S1). These data revealed 1,977 upregulated and 2,109 downregulated genes in WT cells after CL-316,243 treatment. Of these, 349 genes were induced and 449 were downregulated in PPDIVs from WT but not FGF21-KO mice. Among the differentially regulated genes were calmodulins (*Calm1*, *Calm2*, and *Calm3*) (Figure S5A) and the mitochondrial calcium transporter *Slc25a25* and calreticulin *Calr*. We conducted pathway analyses on our RNA-seq data using gene set enrichment analysis (GSEA). Using a targeted approach, we found that the Kyoto encyclopedia of genes and genomes (KEGG) pathway for calcium signaling was enriched in CL-316,243-treated WT compared with KO PPDIVs (Figure S5B). Furthermore, Reactome pathway G-βγ signaling through phospholipase C (PLC) was enriched in CL-316,243 versus vehicle-treated WT but not KO PPDIVs (Figure S5C). These data suggest that FGF21 produces a required second signal consistent with changes in calcium activation of gene expression (Coate et al., 2017). Importantly, not all gene expression changes were dependent on FGF21 because GSEA analyses of the RNA-seq data revealed that BioCarta P38 pathway and Reactome signaling by ILs were induced by CL-316,243 in WT and KO cells (Figure S5D).

Because FGF21 appears to be required for adipocyte browning *in vivo*, we sought to explore the mechanisms in isolated cells. The RNA-seq data suggested that FGF21 is required for control of calcium signaling in adipocytes, and we thus tested the effect of FGF21 treatment on cellular calcium mobilization (Figure 5B). Similar to what has been observed previously in hepatocytes (Chen et al., 2017), FGF21 treatment produced increases in intracellular calcium in PPDIVs, and this effect was blocked by an inhibitor of FGFR tyrosine kinase, PD173074 (Owen et al., 2014; Figure 5B). Next we isolated PPDIVs from WT and FGF21 global KO mice, treated these cells with CL-316,243 for up to 6 h, and measured *Ucp1* and *Dio2* mRNA (Figures 5C and 5D). Although CL-316,243 produced a robust increase in *Ucp1* and *Dio2* mRNA in cells derived from WT animals, the agent was without effect in KO cells, indicating that FGF21 secretion is required for full thermogenic gene induction by cAMP and likely delivers a second signal required for thermogenesis in a cell-autonomous fashion.

We explored the signaling pathways activated downstream of FGF21 by evaluating phosphorylation patterns in PPDIVs derived from iWAT. Addition of FGF21 to these cells increased ERK phosphorylation (Figure 5E), as reported previously (Wu et al., 2010). To explore the possible role of ERK in UCP1 induction, we treated cells with a MEK inhibitor (PD0325901) (Sebolt-Leopold et al., 1999) prior to CL-316,243 (Figures 5F and 5G). Although the MEK inhibitor completely blocked phosphorylation of ERK1 and ERK2 (Figure S5E), the drug had no effect on *Ucp1* or *Dio2* induction (Figures 5F and 5G), revealing that the Ras/ERK pathway is dispensable for thermogenic gene induction.

We then turned our attention to another pathway activated by the FGFR, PLCγ. PLCγ is a substrate of FGFR tyrosine kinases (Huang et al., 2016) and is activated by tyrosine phosphorylation (Patterson et al., 2005). Activation of PLCγ by FGF21 has been observed previously in hepatocytes (Chen et al., 2017). In addition, previous work has shown that FGF21 can promote PLCγ phosphorylation to promote calcium mobilization in exocrine pancreas acinar cells (Coate et al., 2017). Treatment of PPDIVs with FGF21 produced tyrosine phosphorylation of PLCγ within minutes to 1 h of exposure (Figure 5E). To assess the importance of PLCγ in controlling thermogenic gene expression, we pretreated PPDIVs with the PLC inhibitor U-73122 (Macmillan and McCarron, 2010) prior to exposure to CL-316,243 (Figures 5C and 5D). The PLC inhibitor completely blocked induction of *Ucp1* and *Dio2* mRNA in PPDIVs in response to CL-316,243 but had no effect on induction of *Atf3* mRNA (Figure 5H). Interestingly, pre-treatment of PPDIVs with the highly selective calcium chelator BAPTA-AM (Collatz et al., 1997) prior to CL-316,243 also produced a complete block of *Ucp1* mRNA induction but had no effect on induction of *Atf3* (Figures 5I and 5J). These data suggest that, when induced and released by β-adrenergic receptor stimulation, FGF21 activates a PLC/calcium signaling pathway that is required for thermogenic gene expression in response to sympathetic activation of white adipocytes.

## DISCUSSION

FGF21 is a stress-dependent peptide hormone that is synthesized by several organs, including adipose tissue, and has emerged as a novel therapeutic agent to treat obesity and associated metabolic disorders. Although exogenous administration of FGF21 and its analogs has shown beneficial metabolic effects, our understanding of its role in the control of energy homeostasis remains incomplete (Markan and Potthoff, 2016; Tezze et al., 2019). FGF21 is synthesized in numerous cell types in response to stress, including fasting and cold exposure. Among these is the adipocyte, which is known to play an important role in controlling energy homeostasis via regulation of lipid and glucose metabolism and secretion of adipokines, which regulate other metabolically active tissues (Choe et al., 2016; Li et al., 2019). Because fasting increases sympathetic outflow to iWAT, we sought to understand the role of FGF21 in adipose tissue and thus treated mice with the β−3-adrenergic agonist CL-316,243 to specifically target adipocytes, in which the β−3-adrenergic receptor is most highly expressed. Injection of the agonist led to a robust increase in *Fgf21* mRNA and FGF21 protein expression in several tissues, including all adipose depots and liver. Interestingly, targeted deletion of *Fgf21* in the liver completely eliminated the increase in circulating FGF21 in response to β−3-adrenergic receptor activation, indicating that, although hepatocytes do not express the β−3 receptor, activation of this receptor in mice *in vivo* nonetheless leads to increased hepatic mRNA and FGF21 protein expression. Further investigation revealed that this effect was secondary to increased adipocyte lipolysis, leading to production of fatty acids that increase activity of hepatic PPARα, which directly controls FGF21 expression in liver and, therefore, circulating FGF21 levels.

Like chronic cold exposure, repeated injection of β−3-adrenergic agonists leads to browning of iWAT along with increased expression of thermogenic genes (Danysz et al., 2018). Interestingly, FGF21-KO mice were unresponsive to adrenergically activated browning despite no differences in lipolysis *in vivo* or in adipocytes isolated from these mice. More interestingly, despite the fact that circulating FGF21 was not induced in mice with liver-specific KO of FGF21, these mice were sensitive to the browning effects of CL-316,243, suggesting that the circulating hormone is not relevant to this action but, rather, that it is locally produced FGF21 that acts directly on adipocytes as an autocrine factor. Accordingly, mice with targeted deletion of the FGF21 receptor accessory protein β-Klotho were resistant to the beneficial effects of CL-316,243 injection. Furthermore, adipocyte-specific transgenic overexpression of FGF21 effectively promoted browning of iWAT when expressed in WT mice but was without effect in mice lacking β-Klotho. Additional studies *in vivo* and in adipocytes isolated from these mice confirmed that FGF21 delivers a crucial second signal for adipose tissue browning in response to sympathetic activation. It is important to note that, although we relied on expression of *Ucp1* as a marker of iWAT beiging, it is likely that UCP1-mediated uncoupling may not be the sole mechanism of increased energy expenditure because FGF21 has been shown to promote weight loss in UCP1 KO mice (Samms et al., 2015; Véniant et al., 2015). Interestingly, FGF21-mediated iWAT browning appeared to protect UCP1 KO mice housed at room temperature from diet-induced obesity (Keipert et al., 2020). However, it should also be noted that UCP1 KO mice display metabolic abnormalities and do not respond normally to many physiologic parameters.

These findings raise several interesting questions. First, why does adipocyte-secreted FGF21 remain within adipose tissue rather than escaping into the circulation? Unlike other members of the FGF family, FGF21 does not to bind to heparin sulfate, which permits the protein to be secreted into the circulation from hepatocytes (Goetz et al., 2007). It is likely that other factors, perhaps residing in the extracellular matrix of adipose tissue, retain FGF21, enhancing its activity as an autocrine factor. This lack of secretion from adipose tissue is not due to the presence of the FGFR or β-Klotho because other tissues expressing these receptors can release FGF21 into the circulation, and deletion of β-Klotho does not result in a substantial increase in circulating FGF21 in adipocyte-specific transgenic mice.

A second important issue concerns the nature of the signaling pathway initiated by FGF21 in adipocytes that is required for induction of the thermogenic program. RNA-seq experiments using adipocytes derived from FGF21-KO mice treated with CL-316,243 revealed a subset of genes that were not controlled by the β−3-adrenergic agonist in KO cells, providing hints about this signal. Many of these genes are known to be calcium sensitive, leading us to assess calcium pathways in these cells. Indeed, activation of adipocytes with FGF21 led to rapid and sustained tyrosine phosphorylation of PLCγ along with increasesin calcium mobilization, likely resulting from generation of inositol triphosphate. This finding is consistent with our previous work demonstrating that FGF21 triggers intracellular calcium release via an FGFR-PLCγ-inositol triphosphate receptor (IP3R) pathway to stimulate secretion in pancreatic acinar cells (Coate et al., 2017; Lee et al., 2016; Potthoff et al., 2012). Previous studies (Eisenstein and Ravid, 2014; Marcelo et al., 2016; Song et al., 2019; Xu et al., 2017) have also shown that β-adrenergic activation of adipocytes *in vivo* leads to increases in calcium-dependent pathways. These data strongly suggest that these pathways depend on synthesis and secretion of FGF21 to provide and sustain this signal. However, it must be noted that other pathways may also be involved. Although FGF21 has long been known to activate the Ras/MAPK pathway (Tezze et al., 2019; Xu et al., 2016), this effect of the hormone is not required for induction of the thermogenic program. However, there may be other genes, such as *Glut1* (Ge et al., 2011), that are controlled by this pathway and perhaps play a role in the biological effects of the hormone. Nevertheless, the importance of the autocrine actions of FGF21 on adipose tissue provides important insights into how this hormone can best be utilized as a therapeutic agent and suggest that strategies to increase adipocyte FGF21 synthesis might be beneficial.

### Limitations of the study

Although our studies show the requirement of FGF21 for adipose tissue browning in response to repeated injection of a β−3-adrenergic receptor agonist, whether this is also the case during cold exposure or other physiological states of stress remains uncertain. Moreover, the role of FGF21 as an adipose tissue autocrine factor in obese states deserves further investigation.

## STAR★METHODS

### RESOURCE AVAILABILITY

#### Lead contact

Further information for resources and requests should be directed to and will be fulfilled by the lead contact, Alan R. Saltiel, Ph.D. (asaltiel@health.ucsd.edu)

#### Materials availability

Ad-FGF21 transgenic mice used in this study will be made available upon request from the Kliewer/Mangelsdorf lab (davo.mango@utsouthwestern.edu).

#### Data and code availability

RNA-Seq data reported in this paper have been deposited in the National Center for Biotechnology Information (NCBI) Sequence Read Archive (SRA) database, BioProject ID PRJNA725497.

### EXPERIMENTAL MODEL AND SUBJECT DETAILS

#### Animals

Whole body FGF21-KO (Potthoff et al., 2009), liver-specific FGF21-KO (Markan et al., 2014), and adipose-specific β-Klotho-KO (Lan et al., 2017) were described previously. To generate adipose-specific *Fgf21* transgenic mice, a cDNA encoding the mouse FGF21 (Inagaki et al., 2007) was amplified by PCR. A Kozak sequence was added in the front of the ATG start codon. The PCR product was cloned to pBS vector and then the Kozak-Fgf21 was cut with SalI and cloned into the SalI site in the pCAG-Z-EGFP vector (Fukuda et al., 2006). The insert was then cut with *Spe*I/*Afl*II for DNA pronuclear microinjection. The mice were PCR genotyped with primers, GFP - gt2F: TGC CAC CTA CGG CAA GCT GAC C; GFP - gt2R: GGA TCT TGA AGT TCA CCT TGA TGC. The PCR products were 400 bp. CAG-Z-FGF21-EGFP mice were crossed with adipoq-Cre transgenic mice from the Jackson Laboratory (stock# 010803) to generate the adipose-specific transgenic mice, which are GFP+:Cre+. The GFP+:Cre+ transgenic mice were then crossed with floxed β-Klotho mice to generate ABKO/F21Tg mice.

All mice were on C57BL/6J backgrounds. Male littermates were used for all experiments and were fed a standard normal chow (7912, Teklad) and used at 6–8 weeks of age. Prior to IP injection of CL-316,243 (C5976, Sigma-Aldrich) and other drugs, mice were conditioned for 4–5 days by simulating injection with a pencil tip. The dose of CL-316,243 used was 1 mg/kg. Mice were housed in a specific pathogen-free (SPF) facility with 12-h light and 12-h dark cycles and given free access to food and water.

All animal use was approved by the Institutional Animal Care and Use Committee (IACUC) at the University of California, San Diego and the University of Texas Southwestern Medical Center at Dallas, Texas.

#### Cell lines

3T3-L1 preadipocytes (ATCC^,^ CL-173) and *PPDIVS* were cultured and differentiated into adipocyte as described previously (Zhao et al., 2018). See Method details.

Mouse primary hepatocyte cells were grown in William’s medium E (12551–032, Life Technologies) supplemented with 10% of fetal bovine serum (FBS), GlutaMax (35050–061, Life Technologies), and 1% penicillin/streptomycin and plated in collagen-coated plates.

### METHOD DETAILS

#### Free fatty acid (FFA) measurement

FFA concentration was measured using 2 μl serum with the NEFA kit (WAKO). Briefly, 75 μl Reagent A was mixed with the serum, and incubated at 37°C for 5 minutes and then absorbance was measured at 550 nm (reference 660 nm) using the manufacturer’s protocol. The 150 μl Reagent B was added, and incubated at 37°C for 5 minutes, then absorbance was measured again. A standard curve was us to determine sample concentrations.

#### RNA extraction, RT-PCR, and real-time PCR

Liver and WAT RNA was isolated by homogenizing tissues in TRIzol reagent (15596018, Life Technologies) as described by the manufacturer. One microgram RNA was used for cDNA synthesis using the First-Strand cDNA Synthesis Kit (Bio-Rad). Real-time PCR amplification was performed on samples in triplicate with Power SYBR Green PCR Master Mix (Applied Biosystems) using the Applied Biosystems QuantStudio5 real-time PCR System and quantified using an internal standard curve with *Arbp* as the control gene. Primer sequences are listed in Table S1.

#### RNA-sequencing

Wild-type and FGF21-KO primary pre-adipocytes differentiated *in vitro* (PPDIVs) were treated with CL316243 or vehicle for 3 h and RNA was extracted using Pure Link RNA Mini Kit from Ambion. Illumina’s TruSeq RNA Sample Preparation Kit v2 was utilized for library preparation from ~300ng RNA. A 2100 BioAnalyzer (Agilent) preformed library validation followed by sequencing on an Illumina HiSeq 2000 using bar-coded multiplexing at a 100-bp read length. The 100-bp paired-end reads were sequenced to a depth of (2–6) × 10^6^ reads and CASAVA 1.8.2 was used to generate Fastq files. Fastq files were aligned using TopHat2 v2.0.4 and differential gene expression assessed using Cuffdiff v.2.0.2. Data were expressed as fragments per kilobase of exon per million fragments mapped (FPKM).

Pathway analysis of RNaseq results was performed with Gene Set Enrichment Analysis (GSEA_4.0.3). Pathways were retrieved from Reactome, Biocarta and KEGG databases and mouse ENSEMBL_Gene_IDs were used for chip platform. Basic fields were set to default setting with one exception. The metric for ranking genes was set to Diff_of_Classes from Signal2Noise.

#### Histology

Fat and liver tissues were collected and fixed in 10% formalin, then switched in to 70% ethanol. Paraffin embedding and sectioning for H&E staining and IHC was completed at the UCSD Tissue Technology Core. UCP1

#### UCP1 IHC

After baking at 60°C, the tissue cleared and rehydrated, before treatment with Antigen retrieval in Antigen Unmasking Solution (Vector, H-3300) at 95°C for 30 min. The tissue was blocked with Bloxall (Vector, SP-6000) and then 3% Donkey Serum. And incubate with anti-UCP-1 Primary Antibody (Rabbit, Abcam, ab10983, 1:100) for 1 h washed then anti-Rabbit HRP Polymer (Cell IDX, 2RH-100) and washed again before DAB (brown) Chromogen (VWR, 95041–478) and counterstained with Mayer’s Hematoxylin (Sigma, 51275–500ml).

#### H&E staining

After baking at 60°C, the tissue cleared and rehydrated, 5. Before staining with Hematoxylin 1 (Thermo, Cat#7221), followed by Clarifier, Bluing Reagent (Thermo, Cat# 7301) and finally Eosin-Y (Thermo, Cat# 7111).

#### Serum and media-FGF21 measurement

Mice were bled by submandibular puncher using Goldenrod Animal Lancets (MEDIpoint International, Inc), the blood was allowed to clot at room temperature for 30 min prior to serum isolation by centrifugation. Media for FGF21 secretion from 3T3-L1 and PPDIVS was collected from cells following 6-h CL-316,243 treatment in serum free media. Serum and media FGF21 levels were quantified using mouse FGF21 Quantikine ELISA from R&D (MF2100) following the manufacturer’s protocol.

#### Immunoblot

Cells or tissues were lysed by using Nonidet P-40 lysis buffer containing 50 mmol/liter Tris (pH 7.5), 150 mmol/liter NaCl, 10% glycerol, 0.5% Nonidet P-40, and protease inhibitors. The supernatant was used for protein quantification using BCA protein assay kits (23227, Pierce). Proteins were resolved by SDS–PAGE and transferred to nitrocellulose membranes (Bio-Rad).

Antibodies used: Rabbit polyclonal anti-UCP1 antibody (ab10983, Abcam), rabbit polyclonal anti-DIO2 (ab135711, Abcam), polyclonal goat anti-FGF21 (AF3057, R&D), rabbit polyclonal anti-phospho-p44/42 MAPK (Erk1/2) (Thr202/Tyr204) (#9101, Cell Signaling), anti-p44/42 MAPK (Erk1/2) (137F5) rabbit mAb (#4695, Cell Signaling), rabbit anti-phospho-PLCγ1 (Tyr783) antibody (#2821, Cell Signaling), and rabbit anti-PLCγ1 (D9H10) XP Rabbit mAb (#5690, Cell Signaling).

#### Tissue culture

3T3-L1 preadipocytes (ATCC^,^ CL-173) and *PPDIVS* were cultured and differentiated into adipocyte as described previously (Zhao et al., 2018). Briefly, cells were grown to full confluence in DMEM (GIBCO) with 10% Fetal Bovine Serum FBS, 100 U/ml penicillin, and 100 μg/ml streptomycin. Cells were grown in a 37°C humid incubator with 5% CO_2_, then switched into differentiation media supplemented with 1 μM dexamethasone, 0.5 mM 3-isobutyl-methylxanthine, and 1 μg/ml insulin (MDI). The MDI cocktail was used to induce 3T3-L1 differentiation for the first 4 days followed by addition of insulin for 3 more days. After 48 h, the media was then replaced to regular DMEM. For CL-316,243 treatment the media was changed to serum deprived media for 3 h followed by drug treatment for an additional 3 and 6 h.

Preadipocytes were isolated from iWAT of 4–6 weeks old male mice. The iWAT was finely minced and digested with 1 mg/ml collagenase (C6885, Sigma) in serum free DMEM. The minced fat was then incubated for 20–30 min at 37°C with gentle agitation. To neutralize the collagenase, FBS was added to 10%, the mixture was filtered through a 100 μm filter and centrifuged at 1500 rpm for 5 min; the supernatant was aspirated and the pellet was washed and then the cells cultured in 10 cm tissue culture plates in DMEM media including 15% FBS. After one passage, the preadipocytes were grown to confluence and then two days later induced to differentiate with 500 μM 3-isobutyl-1-methylxanthine, 250 nM dexamethasone, 1μg/mL insulin and 1μM troglitazone for 4 days, followed by insulin for 3 days. Cells were used for experiments at 8 or 9 days after the initiation of differentiation.

Primary hepatocytes were isolated from 6 week old male mice as described previously (Smets et al., 2002). Briefly mice were anesthetized and a midline laparotomy performed. The inferior vena cava was cannulated and perfused at a rate of 2.5 ml/min. The portal vein was sectioned, and the solution allowed to flow through the liver. The liver was perfused sequentially with calcium-free HEPES-phosphate buffer (pH 7.4) followed by 40 μg/ml of Liberase TM (5401127001, Roche) solution. Then the liver was washed in HEPES phosphate buffer (Calsuim-free). Hepatocytes were removed by mechanical dissociation of the liver tissue, filtered through sterile 100 μm mesh nylon (Scientifics, Frederick, MD), washed twice by centrifugation at 50*g* for 5 min. Dead cells were removed by 36% percoll density gradient centrifugation at 100 × g for 10 min. Cells were resuspended with William’s medium E (12551–032, Life Technologies) supplemented with 10% of fetal bovine serum (FBS), GlutaMax (35050–061, Life Technologies), and 1% penicillin/streptomycin and plated in collagen-coated plates. Five h later, the cells were treated with 10 μM isoproterenol for 4 h, and then the media was collected and FGF21 secretion was measured by ELISA as described above.

#### Calcium mobilization assay

For Fura2-AM experiments, PPDIVs cells were preincubated with 0.5 μmol/L Fura2-AM (F1221, Thermofisher) for 15 min at 37°C before imaging. Cells were washed twice with modified HBSS (1 × HBSS with 2 g/l glucose, pH 7.4, made from 10 × HBSS (14065, GIBCO)) and imaged in the dark at room temperature. Images were acquired under a Zeiss Observer Z1 microscope equipped with a 40x/1.3NA objective and Photometrics Evolve 512 EMCCD. Fura2 dual excitation ratio imaging involved 2 excitation filters, ET340x and ET380x for 340 and 380 nm excitation, and an HQ535/45 m emission filter. Exposure times were 200 ms, and images were taken every 30 s. Imaging data were analyzed with Metafluor 7.7 software (Molecular Device). Fluorescence images were background-corrected by deducting the background (regions with no cells) from the emission intensities of cells loaded with Fura2-AM. The 340/380-nm emission ratio was calculated at different times. Traces were normalized by setting the emission ratio to 1 before the addition of drugs.

### QUANTIFICATION AND STATISTICAL ANALYSIS

Statistical significance was determined using either a Student’s t test (when the exerpimental designed contained only one variable) or two-way ANOVA with a Holm-Sidak post hoc analysis to adjust for multiple comparisons when the experimental design was two factorial. Sample sizes, experimental replicates, and specific statistical test used are described in the Figure Legends. A multiplicity adjusted significance threshold of p ≤ 0.05 was used throughout the study. Error bars indicate s.e.m.

## Supplementary Material

1

2

## Figures and Tables

**Table:** KEY RESOURCES TABLE

REAGENT or RESOURCE	SOURCE	IDENTIFIER
Antibodies		
Rabbit polyclonal anti-UCP1 antibody	Abcam	Cat# ab10983; RRID:AB_2241462
Rabbit polyclonal anti-DIO2 antibody	Abcam	Cat# ab135711; RRID:AB_2892107
Polyclonal goat anti-FGF21	R&D	Cat# AF3057; RRID:AB_2104611
Rabbit polyclonal anti-phospho-p44/42 MAPK (Erk1/2) (Thr202/Tyr204)	Cell Signaling	Cat# 9101; RRID:AB_331646
Anti-p44/42 MAPK (Erk1/2) (137F5) rabbit mAb	Cell Signaling	Cat# 4695; RRID:AB_390779
Rabbit anti-phospho-PLCγ1 (Tyr783) antibody	Cell Signaling	Cat# 2821; RRID:AB_330855
Rabbit anti-PLCγ1 (D9H10) XP Rabbit mAb	Cell Signaling	Cat# 5690; RRID:AB_10691383
Chemicals, peptides, and recombinant proteins		
CL 316,243 hydrate	Sigma-Aldrich	Cat# C5976
SB-203,580	Sigma-Aldrich	Cat# S8307
H 89 dihydrochloride	TOCRIS	Cat# 2910
PD98059	Selleck Chemicals	Cat# S1177
PD173074	Sigma-Aldrich	Cat# P2499
PD 0325901	Sigma-Aldrich	Cat# PZ0162
U-73122	Sigma-Aldrich	Cat# U6756
BAPTA-AM	ThermoFisher	Cat# B1205
ATGL Inhibitor, Atglistatin	Sigma-Aldrich	Cat# 5.30151
Recombinant mouse FGF21 Protein	R&D Systems	Cat# 8409-FG-025/CF
Critical commercial assays		
NEFA kit	WAKO	Cat# 999–34691, 995–34791, 993–35191, 276–76491
FGF21 Quantikine ELISA	R&D	Cat# MF2100
Mouse IL-6 Quantikine ELISA Kit	R&D	Cat# SM6000B
cAMP Enzyme Immunoassay Kit	Sigma-Aldrich	Cat# CA201
Deposited data		
RNA-Sequencing analysis of WT and FGF21-KO PPDIVs treated with or without CL-316,243	This paper	SRA: PRJNA725497
Experimental models: Cell lines		
3T3-L1 preadipocytes	ATCC	CL-173
Primary preadipocytes	C57Bl6 iWAT	NA
Mouse primary hepatocyte	C57Bl6 liver	NA
Experimental models: Organisms/strains		
Mouse: whole body FGF21-KO	Potthoff et al., 2009	NA
Mouse: liver-specific FGF21-KO (LFKO)	Markan et al., 2014	NA
Mouse: adipose-specific β-Klotho-KO (ABKO)	Lan et al., 2017	NA
Mouse: adipose-specific FGF21 Tg	This paper	NA
Oligonucleotides		
25 nmole DNA Oligo, Standard Desalting (primers sequences are listed in Table S1)	IDT	N/A
Software and algorithms		
Prism 6.0	GraphPad	6.0

## Highlights

- Catecholamines stimulate adipocyte FGF21 expression
- Adipocyte FGF21 is required for full stimulation of thermogenic gene expression
- Adipocyte lipolysis increases hepatic FGF21 expression and circulating FGF21
- Circulating FGF21 is dispensable for white adipose tissue browning
