# Supplementary material for: FGF21 promotes thermogenic gene expression as an autocrine factor in adipocytes
Source: Cell Rep. Author manuscript; Available in PMC 2021 Jul 21. (PMC8293281; doi:10.1016/j.celrep.2021.109331)
Supplement: 1 [file NIHMS1720099-supplement-1.pdf]

Cell Reports, Volume *35*

Supplemental information

FGF21 promotes thermogenic gene expression as an autocrine factor in adipocytes

Mohammad Abu-Odeh, Yuan Zhang, Shannon M. Reilly, Nima Ebadat, Omer Keinan, Joseph M. Valentine, Maziar Hafezi-Bakhtiari, Hadeel Ashayer, Lana Mamoun, Xin Zhou, Jin Zhang, Ruth T. Yu, Yang Dai, Christopher Liddle, Michael Downes, Ronald M. Evans, Steven A. Kliewer, David J. Mangelsdorf, and Alan R. Saltiel

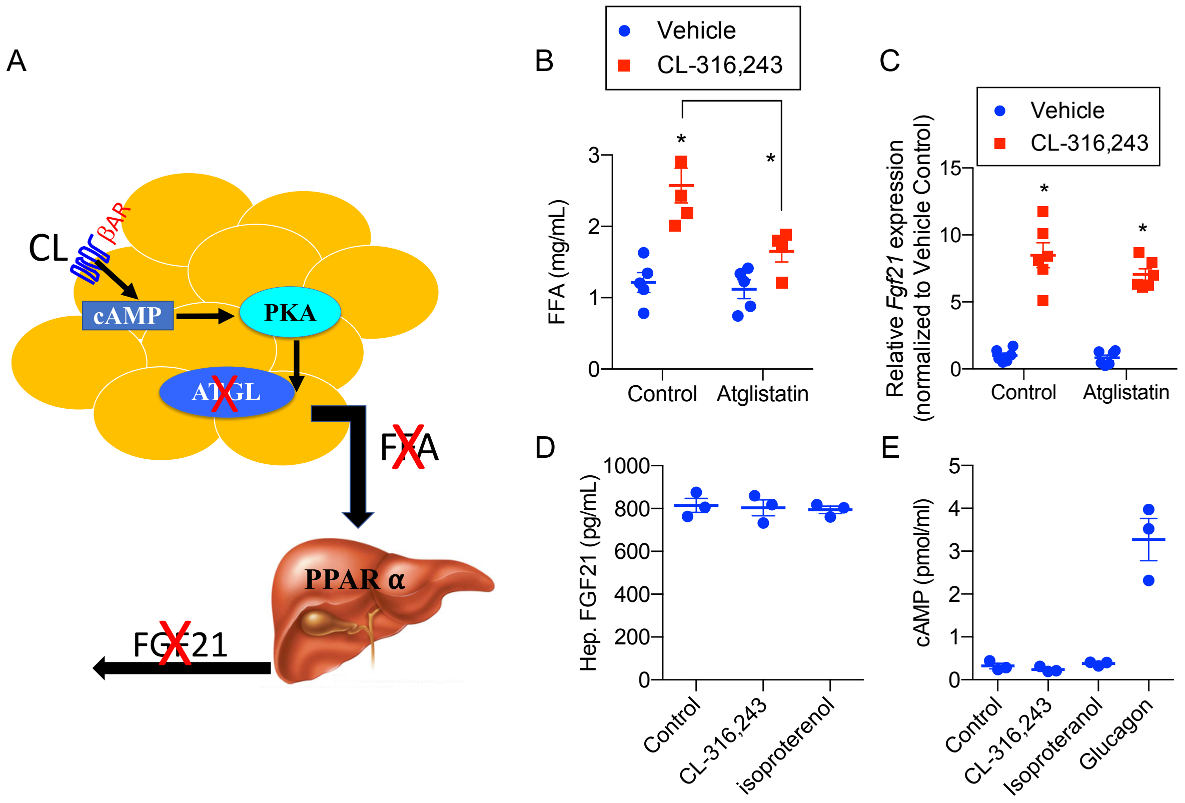

Figure S1. Crosstalk between adipose tissue and liver, Related to Figure 2. (A). Experiment design for the cross talk between adipose tissue and liver mediated by FFA. (B) Serum FFA from mice pretreated with ATGL inhibitor administered intraperitoneally for 30 minutes followed by IP injection of 1 mg per kg (body weight) CL-316,243 or vehicle for 4 h, $n = 5$ mice per croup. (C) iWAT-FGF21 mRNA-expression isolated from mice used in (B). (D) FGF21 secretion to the media from primary hepatocyte following 6h treatment of CL-316,243 or isoproterenol. (E) primary hepatocyte-cAMP following treatments the cells with CL-316,243 10µM, isoprotenol 10µM, and Glucagon 10nM. * p-value < 0.05 from Holm-Sidak post hoc analysis after significant two-way ANOVA for vehicle versus CL-316,243 unless otherwise indicated with line

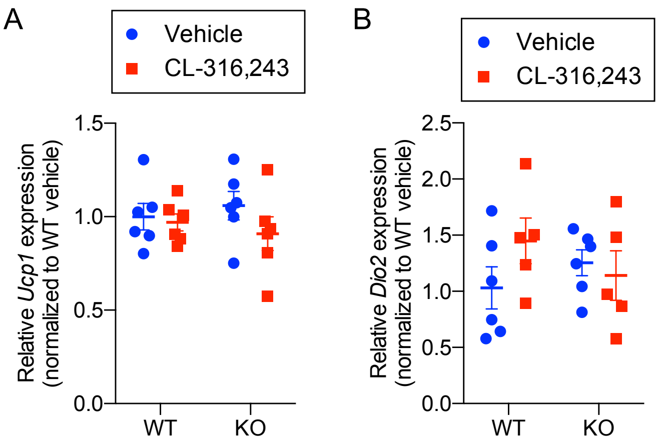

Figure S2. Thermogenic gene expression in BAT, Related to Figure 3. (A) *Ucp1* and (B) *Dio2* expression in the BAT of WT and whole body FGF21-KO mice, treated with 1 mg/kg CL-316,243 or vehicle daily for 7 days, $n = 6$ in each group. No significant differences detected by two-way ANOVA.

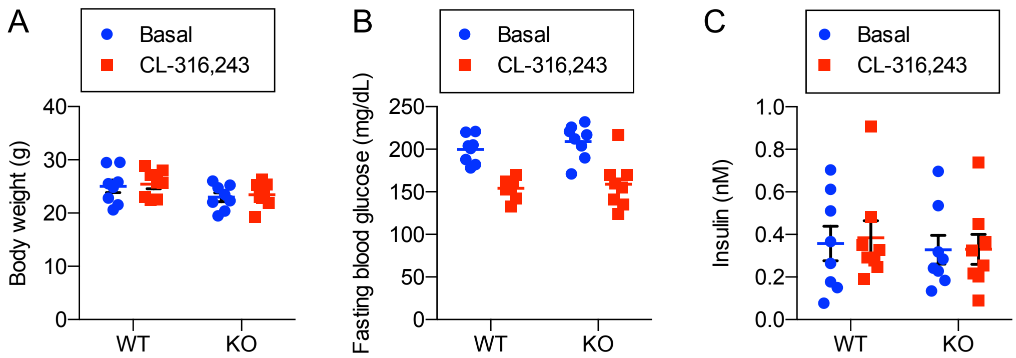

Figure S3. Metabolic phenotype of WT and FGF21-KO mice treated with CL-316,243 for 7 days, Related to Figure 3. WT and whole body FGF21-KO mice, treated with 1 mg/kg CL-316,243 daily, n = 8 mice per genotype. (A) Bodyweight at baseline and after 7 days of treatment. (B) Fasting blood glucose at baseline and after 7 days of treatment. (C) Fasting serum insulin at baseline and after 7 days of treatment.

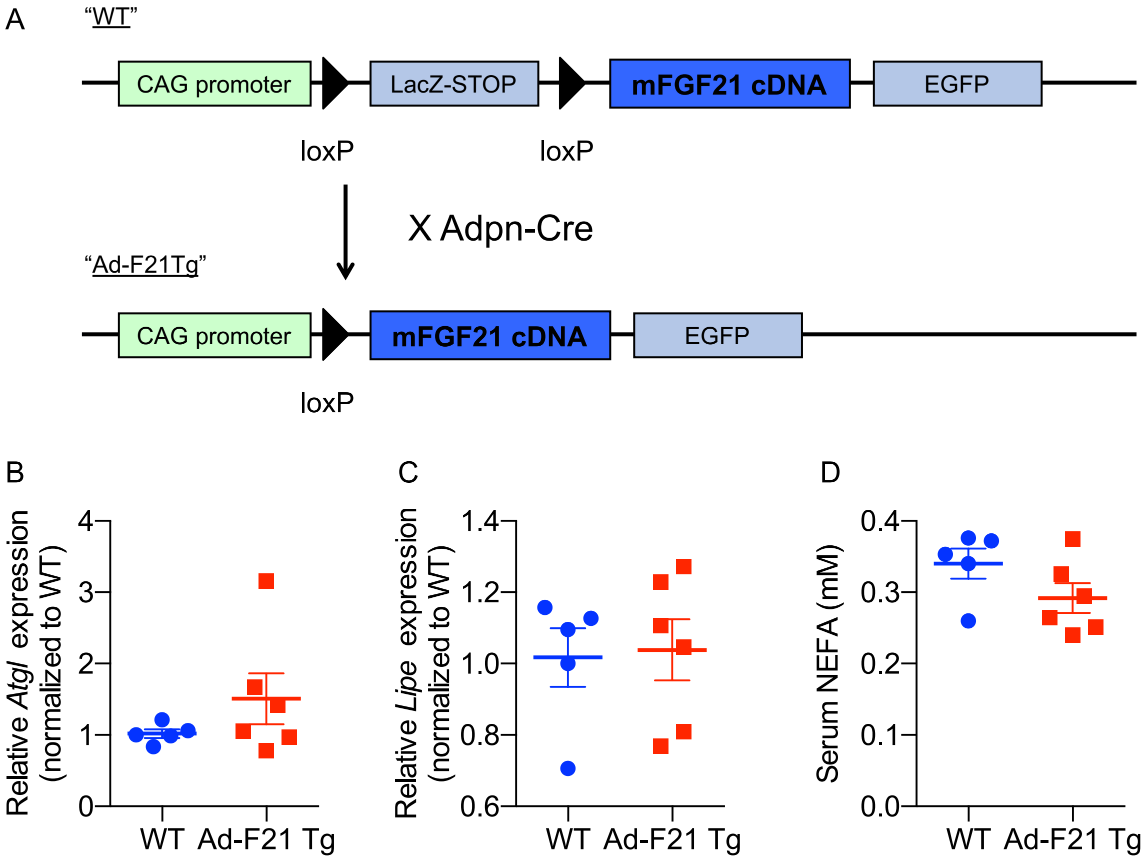

Figure S4. Lipolysis genes and FFA levels in adipose FGF21 Tg mice, Related to Figure 4. (A) Experimental design to generate adipose specific Fgf21 transgenic (Ad-F21 Tg) mice. (B) iWAT *Atgl*-mRNA expression. (C) iWAT *Lipe*-mRNA expression. (D) FFA serum levels. n = 5-6 mice/genotype.

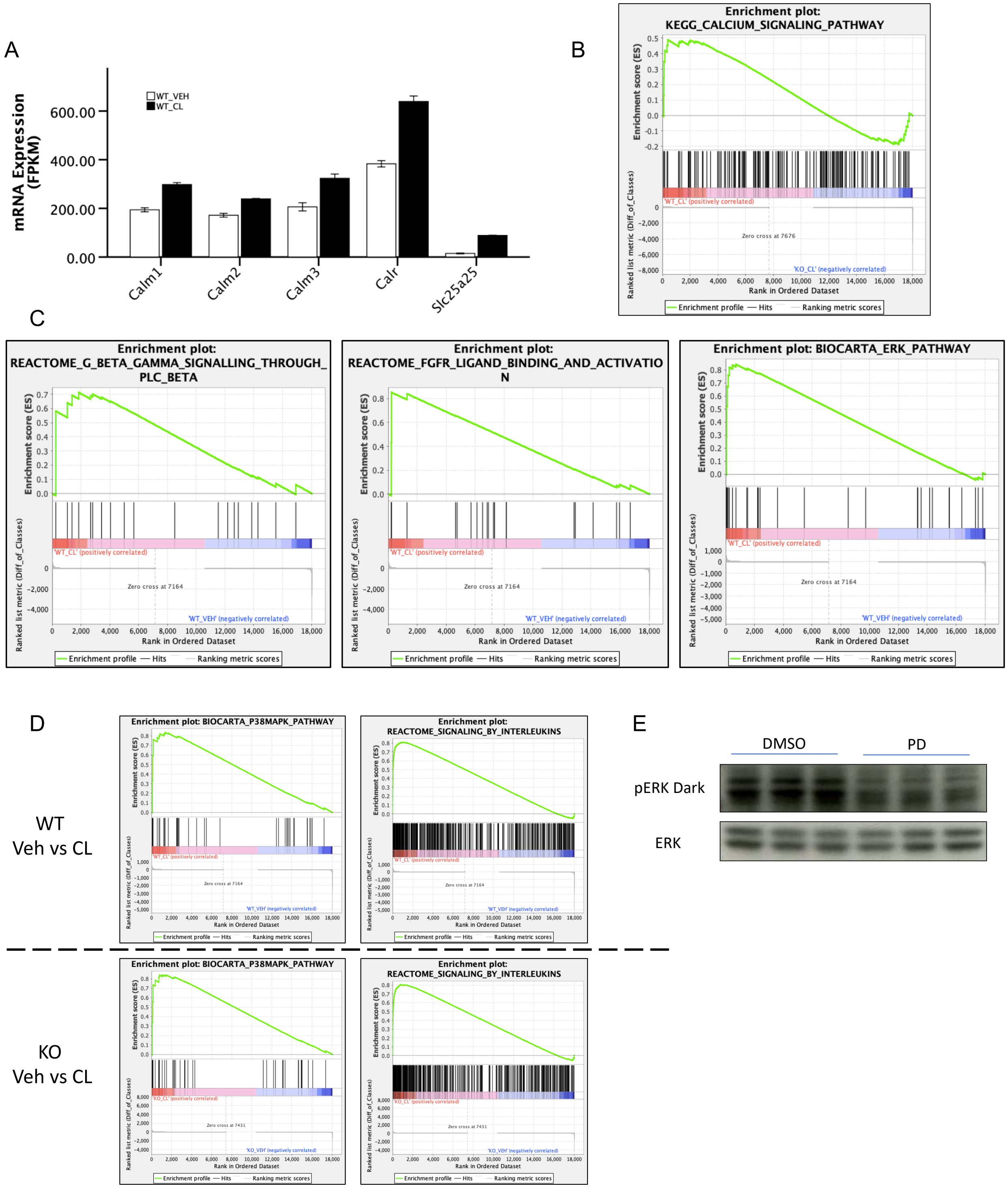

Figure S5. Genes involved in calcium signaling are upregulated by CL-316,243 in a FGF21 dependent manner, Related to Figure 5. (A) Calcium handling gene expression from RNA sequencing data preformed on WT and FGF21-KO PPDIVs treated with vehicle or 1 μM CL-316,243 (CL). (B and C) Pathway analyses of RNA sequencing data using Gene Set Enrichment Analysis. (D) Pathways enriched with CL treatment that do not change with FGF21 Knockout. (E) Representative WB that for the validation of MEK inhibitor on the phosphorylation of ERK. **p*-value < 0.0 from student's t-test versus baseline.

Table S1: qPCR primer sequences, Related to Figures 1-4.

Primer	Forward	Reverse
Arbp	5’-CACTGGTCTAGGACCCGAGAA-3’	5’-AGGGGGAGATGTTCAGCATGT-3’
Atf3	5’-GABGATTTTGCTAACCTGACACC-3’	5’-TTGACGGTAACTGACTCCAGC-3’
Ucp1	5’-AGGCTTCCAGTACCATTAGGT-3’	5’-CTGAGTGAGGCAAAGCTGATTT-3’
FGF21	5’-GTGTCAAAGCCTCTAGGTTTCTT-3’	5’-GGTACACATTGTAACCGTCCTC-3’
Pgc1a	5’-CCACTTCAATCCACCCAGAAAG-3’	5’-TATGGAGTGACATAGAGTGTGCT-3’
Adrb3	5’-GGCCCTCTCTAGTTCCCAG-3’	5’-TAGCCATCAAACCTGTTGAGC-3’
Il6	5’-TAGTCCTTCCTACCCCAATTTCC-3’	5’-TTGGTCCTTAGCCACTCCTTC-3’
Dio2	5’-AATTATGCCTCGGAGAAGACCG-3’	5’-GGCAGTTGCCTAGTGAAAGGT-3’
Dusp4	5’-ACCACAAGGCCGACATCAG-3’	5’-GTCCTTTACTGCGTCGATGTACTC-3’
